# Interlobar Empyema

**Published:** 1905-02-18

**Authors:** 


### INTERLOBAR EMPYEMA.

There are few conditions on occasion more easy, and on occasion more difficult of diagnosis than empyema. In an uncomplicated case the extensive dulness and displacement of the heart admit of no dubious interpretation, but there are three varieties of the lesion which at times almost defy diagnosis. These are the interlobar, the diaphragmatic, and the loculated types.

Sir William Broadbent offers some interesting observations on the interlobar variety. In the first place, he considers that every empyema spontaneously discharging by way of a bronchus is presumptively interlobar, on the ground that an effusion arising between two lobes of the lung finds the line of least resistance in the direction of the large tubes at the root of the lung, and is thus favourably situated for eroding a large air-tube and escaping by the trachea. This is an ingenious theory and has much to recommend it, for, as the author observes, the natural tendency of an effusion, free in the pleural cavity, is to flatten the lung against the mediastinum.

Two cases are quoted to illustrate the clinical side of the condition. The first was a man of 60 who, after passing through an attack of influenza, relapsed, and developed signs pointing to a lesion of the right chest. These signs were slight impairment of percussion resonance, and areas of diminished breathing, both varying from day to day, and shifting from the upper lobe to the lower and back again. Finally the dulness was located at the apex of the axilla, and exploration with a needle revealed the presence of pus. The cavity was drained, and the patient made a complete and unusually rapid recovery.

The second case was that of a boy of 10, who underwent a frank attack of lobar pneumonia in the left side, both lobes being consecutively involved. A crisis occurred on the ninth day, but the temperature remained normal for one day only, and then became of a hectic, swinging type. The physical signs were very variable as regards auscultation and percussion, but there was a progressive displacement of the heart to the right. Finally, there was absolute dulness of the left base behind as high as the angle of the scapula, with absence of vocal fremitus, and with marked ægophony. Exploratory puncture below the angle of the scapula was without result on several occasions. Finally, in view of the variability in the signs an interlobar empyema was suspected, and puncture in the anterior axillary line just below the lower border of the pectoralis major resulted in the withdrawal of pus. Complete recovery followed the evacuation of the abscess.

The points that guide the diagnosis are the history, which often tells of an antecedent lesion of the lung, associated with a swinging fever, and a daily variation in the character of the physical signs.

